# NR1D1 regulation by Ran GTPase via miR4472 identifies an essential vulnerability linked to aneuploidy in ovarian cancer

**DOI:** 10.1038/s41388-021-02082-z

**Published:** 2021-11-06

**Authors:** Zied Boudhraa, Kossay Zaoui, Hubert Fleury, Maxime Cahuzac, Sophie Gilbert, Guergana Tchakarska, Jennifer Kendall-Dupont, Euridice Carmona, Diane Provencher, Anne-Marie Mes-Masson

**Affiliations:** 1 Centre de recherche du Centre hospitalier de l’Université de Montréal (CRCHUM), Montreal, QC Canada; 2 Institut du cancer de Montréal (ICM), Montreal, QC Canada; 3 Division of Gynecologic Oncology, Université de Montréal, Montreal, QC Canada; 4 Department of Medicine, Université de Montréal, Montreal, QC Canada

**Keywords:** Ovarian cancer, Tumour heterogeneity

## Abstract

While aneuploidy is a main enabling characteristic of cancers, it also creates specific vulnerabilities. Here we demonstrate that Ran inhibition targets epithelial ovarian cancer (EOC) survival through its characteristic aneuploidy. We show that induction of aneuploidy in rare diploid EOC cell lines or normal cells renders them highly dependent on Ran. We also establish an inverse correlation between Ran and the tumor suppressor NR1D1 and reveal the critical role of Ran/NR1D1 axis in aneuploidy-associated endogenous DNA damage repair. Mechanistically, we show that Ran, through the maturation of miR4472, destabilizes the mRNA of NR1D1 impacting several DNA repair pathways. We showed that NR1D1 interacts with both PARP1 and BRCA1 leading to the inhibition of DNA repair. Concordantly, loss of Ran was associated with NR1D1 induction, accumulation of DNA damages, and lethality of aneuploid EOC cells. Our findings suggest a synthetic lethal strategy targeting aneuploid cells based on their dependency to Ran.

## Introduction

Ovarian cancer is the most lethal gynecologic malignancy in North America, with a 5-year survival rate of 45% [1]. The most common form is epithelial ovarian cancer (EOC) where ~60% of patients present with a high-grade serous carcinoma (HGSC) [2]. Extensive genome sequencing studies have revealed that HGSC presents extremely high intra-tumoral heterogeneity [3], which poses specific challenges for therapeutic strategies. Within a tumor, some cell populations may be drug-resistant (or become drug-resistant), leading to patient relapse. However, independent of their heterogeneity, these EOC cell populations have complex karyotypes and aneuploidy [3, 4]. Aneuploidy is a main enabling characteristic of cancer cells allowing them to acquire eight biological capabilities termed the “hallmarks of cancer,” which lead to cell survival, proliferation, and dissemination [5]. However, aneuploidy itself generates specific vulnerabilities, which cancer cells need to overcome (reviewed in [6]). Therefore, a strategy that specifically targets aneuploidy may provide a promising approach for treating EOC, including cases that are carboplatin resistant.

The small GTPase Ran (Ras-related nuclear protein) belongs to the Ras family of GTPases. Its role in tumor initiation and progression has been investigated in several tissues [7]. We have shown that Ran GTPase is a promising candidate biomarker of EOC with therapeutic value [8–10]. More importantly, it has been shown that Ran silencing has little or no effect in a range of normal cells suggesting that this GTPase is an attractive therapeutic target [11, 12]. The nuclear receptor subfamily 1 group D member 1 (NR1D1) is a transcriptional repressor of the circadian core clock components [13] and of genes involved in metabolism and inflammation [14, 15]. NR1D1 is now recognized as a tumor suppressor since it was shown to abrogate tumor cell proliferation, lipid metabolism, autophagy, and DNA damage response (DDR) in several cancer types [16–19].

Here we investigated the mechanisms behind the selective sensitivity that cancer cells have to Ran knockdown (KD) in comparison to normal cells. We report that Ran dependency in EOC cells is governed by ploidy, genomic instability, and spontaneous DNA damage. Mechanistically, Ran modulates different DNA repair pathways and supports cell survival of aneuploid EOC cells by destabilizing the mRNA of the tumor suppressor NR1D1 via miR4472. Furthermore, we demonstrated that NR1D1 was able to interact with both PARP1 and BRCA1 leading to the inhibition of their related DNA repair pathways. Collectively, our study is the first to report a link between Ran/NR1D1 axis and aneuploid EOC cell survival. We therefore suggest a synthetic lethal strategy targeting aneuploid cells based on their dependency to Ran.

## Results

### Ran dependency is associated with aneuploidy

**Fig. 1:**
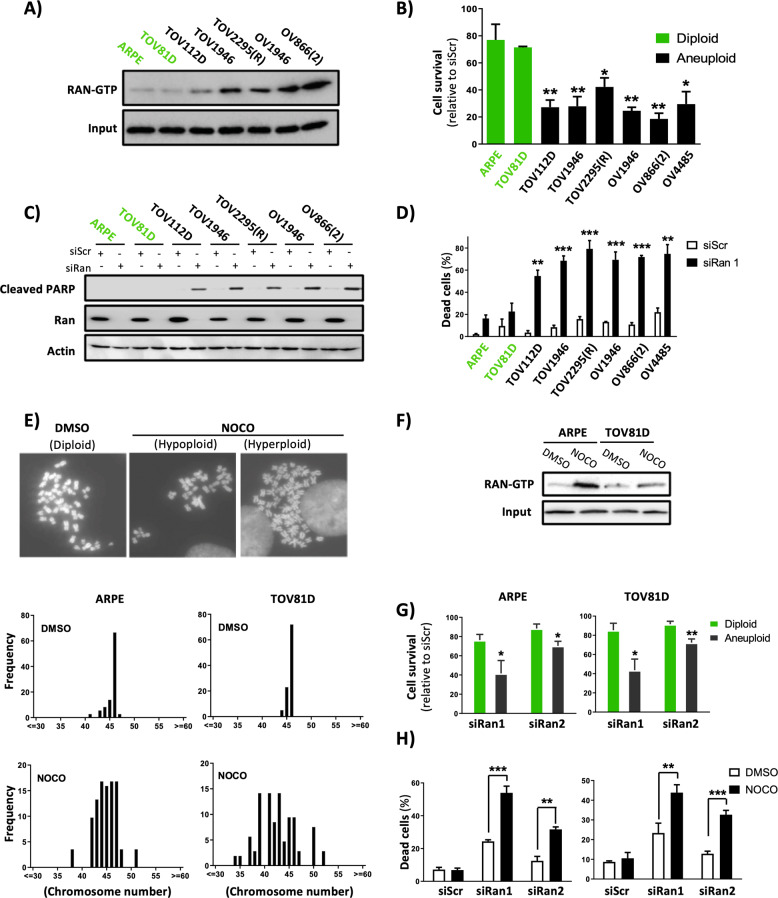
Ran dependency in EOC cell lines is associated with aneuploidy. **A** Ran activity (Ran-GTP) was evaluated in EOC cells and normal ARPE cells using Ran-GTP pull-down assay. Diploid cells in green. **B** EOC cells and normal ARPE cells were transfected with siRan1 and subjected to proliferation assays using an IncuCyte live cell monitoring system. Green and black bars represent diploid and aneuploid cells, respectively. Data are expressed as the percentage of siScr-transfected cells at the end of experiment (96 h post transfection) and are representative of at least three independent experiments. **C**, **D** Cells were transfected with siRan1 in order to evaluate apoptosis (96 h post transfection) by western blot using an anti-cleaved PARP antibody (**C**) and by flow cytometry using an Annexin V fluorescent antibody (**D**). The percentage of Annexin V-positive cells (means from three independent experiments) is shown. **E** Aneuploidy was induced in ARPE and TOV81D cells by nocodazole treatment and was assessed by metaphase spread experiments. Upper panel: representative images of diploid, hypoploid, and hyperploid cells obtained after nocodazole treatment. Lower panel: quantification of chromosome number per cell (chromosomes were counted in at least 100 nuclei per condition). **F** Cells were treated as in (**E**) and Ran activity was assessed by Ran-GTP pull-down assays. **G**, **H** Aneuploidy was induced in ARPE and TOV81D cells by nocodazole treatment prior to Ran KD with siRan1 and siRan2. Subsequently, cell proliferation (**G**) and apoptosis (**H**) were assessed as in (**B**) and (**D**), respectively. *P < 0.05, **P < 0.01, ***P < 0.001 (*n* ≥ 3, Student’s *t*-test). In **B**, *t*-tests compared the indicated cancer cells with ARPE cells. In **D**, *t*-tests were among siRan1-transfected cells, comparing indicated cancer cells with ARPE cells.

We first performed a systematic study on the activity of Ran in five aneuploid HGSC cell lines [TOV1946, TOV2295(R), OV1946, OV866(2), and OV4485], one aneuploid endometrioid EOC cell line (TOV112D), and one diploid low-grade serous EOC cell line (TOV81D), together with the non-transformed diploid ARPE cell line (see ploidy state in Table 1). Irrespective of their subtype, grade, or origin, Ran-GTP pull-down assays showed that all aneuploid cells displayed a higher activity of Ran than the diploid cells (Fig. 1A and Supplementary Fig. S1A). Ran activity relies on a specific guanine nucleotide exchange factor (GEF) that promotes the GTP loading of Ran [7]. Since the only known RanGEF is the regulator of chromosome condensation 1 (RCC1) [7, 20], we investigated the expression of this protein in our cell lines. In accordance with the detected higher Ran activity, we observed that aneuploid cells harbored an increased expression of RCC1 (Supplementary Fig. S1B). Furthermore, unlike diploid cells, Ran KD using two different siRNAs (siRan1 or siRan2) (Supplementary Fig. S1C) decreased cell viability in all aneuploid EOC cell lines (Fig. 1B and Supplementary Fig. S1D), regardless of their sensitivity to carboplatin (Table 2). We also found that Ran KD induced apoptosis specifically in aneuploid EOC, without any significant effect in diploid cells, as assessed by cleaved PARP and Annexin V-positive cells (Fig. 1C, D and Supplementary Fig. S1E, F). Collectively, our data indicate that a differential sensitivity between diploid (normal or cancer) and aneuploid EOC cells can be defined by the loss of Ran.

**Table 1:** Ploidy state of the cell lines used in this study.

Cell line	Chromosome number (range)^a^
ARPE	46
TOV81D	46
TOV112D	49–54
TOV1946	51–62
OV1946	57–65
TOV2295(R)	42–65
OV866(2)	61–89
OV4485	51–70

^a^Determined by metaphase spread experiments.

**Table 2:** Carboplatin sensitivity of the EOC cell lines used in this study.

Cell line	Carboplatin IC_50_^a^ (μM)	Reference
TOV112D	13.4	Letourneau et al. [36]
OV866(2)	32.1 ± 7.1	Fleury et al. [35]
TOV1946	5.2 ± 1.1	This study
OV1946	3.4 ± 0.2	Brodeur et al. [34]
TOV2295(R)	0.93 ± 0.01	Letourneau et al. [36]
OV4485	6.1 ± 0.3	Fleury et al. [35]

^a^Determined by clonogenic assays.

To further examine the association between aneuploidy and sensitivity to the loss of Ran, we induced aneuploidy in ARPE and TOV81D cells with nocodazole [21]. Our metaphase spread experiments showed heterogeneous cell populations with aberrant chromosome numbers after nocodazole treatment (Fig. 1E). Interestingly, we observed that induction of aneuploidy was accompanied by an increase in RCC1 expression (Supplementary Fig. S1G) and Ran activity (Fig. 1F), indicating that Ran activity and aneuploidy are closely related and that the activation of Ran is supported by an induction of RCC1. Furthermore, aneuploid ARPE and TOV81D cells became more sensitive to the loss of Ran, demonstrating impaired cell proliferation (Fig. 1G) and induction of apoptosis (Fig. 1H). To corroborate this finding, we induced aneuploidy (tetraploidy) with an alternative method using the inhibitor of cytokinesis cytochalasin D (Supplementary Fig. S1H). As with nocodazole treatment, induction of tetraploidy sensitized cells to Ran KD (Supplementary Fig. S1I). Overall, our results strongly suggest that the higher activity of Ran in aneuploid cancer cells is a consequence of chromosomal instability and that Ran dependency is associated with the ploidy rather than the reported “normal/cancer” state of cells [11, 12]. Therefore, the known function of Ran in cancer initiation and progression [7] might be associated with its role in aneuploidy.

### Ran is involved in DNA repair systems

**Fig. 2:**
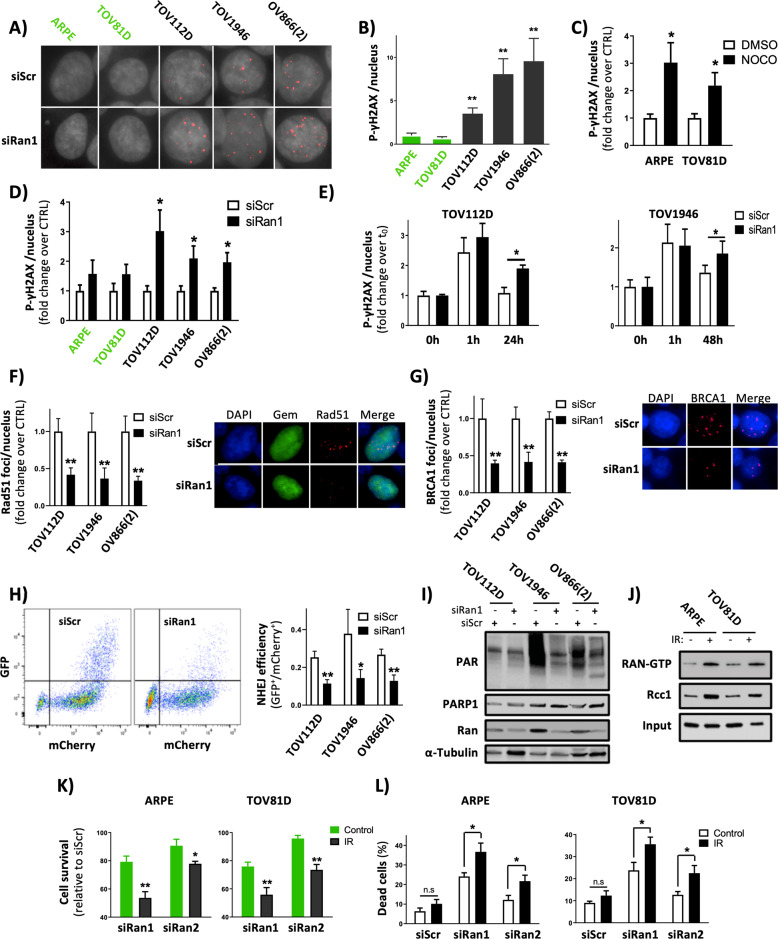
Ran is involved in DNA repair and cell survival after DNA damage. **A** Representative images of p-γH2AX (red) foci in normal ARPE and EOC cancer cells following Ran KD. **B**–**D** Quantitative analysis of p-γH2AX foci in normal and EOC cancer cells (**B**); in ARPE and TOV81D cells after induction of aneuploidy with nocodazole (NOCO) (**C**); and in diploid and aneuploid cells after Ran KD (**D**). **E** TOV112D and TOV1946 cells were transfected with siRan1 and exposed to 2 Gy X-rays. Cells were fixed at the indicated recovery time points and immunostained for p-γH2AX. Quantitative analysis of p-γH2AX foci is shown. **F**–**G** Representative images of Rad51 (**F**) and BRCA1 (**G**) immunostaining (red) 1 h after 10 Gy irradiation in cells transfected with siRan1. Green: geminin, Blue: DAPI. Quantitative analysis of BRCA1 and Rad51 foci (right panels) are shown. **H** NHEJ efficiency was measured in TOV112D, TOV1946, and OV866(2) cells that were co-transfected with a NHEJ reporter plasmid (containing I-SceI restriction site), I-SceI endonuclease, and mCherry expression vectors. The ratio of GFP-positive cells versus that of mCherry-positive cells was determined by flow cytometry. **I** EOC cells were transfected with siScr or siRan1 and pre-treated with H_2_O_2_. PARylated proteins were analyzed by western blotting using anti-Par antibody 72 h after transfection. **J** ARPE and TOV81D cells were exposed to 0.5 Gy X-rays before assessing Ran activity and RCC1 expression. **K**, **L** ARPE and TOV81D cells were transfected with siScr, siRan1, or siRan2 and then exposed to 0.5 Gy X-rays in order to assess cell proliferation (**K**) and apoptosis (**L**). *P < 0.05, **P < 0.01, ***P < 0.001 (*n* ≥ 3, Student’s *t*-test). In **B**, *t*-tests compared the indicated cancer with ARPE cells.

One of the characteristics differentiating aneuploid cancer cells from other cells is the imbalance between sources of DNA damage and systems that control genome integrity. To determine the basal levels of DNA damage in our aneuploid and diploid cell lines, we carried out analyses of p-ɣH2AX foci, a known marker for DNA double-strand breaks (DSBs). Our results showed that the aneuploid cancer cells exhibited enhanced p-ɣH2AX foci numbers when compared to the diploid cells (Fig. 2A, B). To rule out the possibility that this observation was due to a difference in cell-doubling time and replicative stress, we synchronized the cells in G1 phase by serum starvation and the same results as asynchronous cells were observed (Supplementary Fig. S2A). More interestingly, when aneuploidy was induced in diploid ARPE and TOV81D cells, the number of p-ɣH2AX foci was significantly increased (Fig. 2C). These results indicate that the presence of spontaneous DNA damage is one of the characteristics of aneuploid cells.

To investigate if any association exists between the loss of Ran and DNA damage accumulation, cells were transfected with siRan1 or siRan2 before p-ɣH2AX foci analysis. We showed that, in contrast to diploid cells, Ran KD induced an accumulation of p-ɣH2AX foci in aneuploid cells (Fig. 2D and Supplementary Fig. S2B) and raised the possibility that Ran is involved in DDR pathways. To determine this, we induced DNA DSBs by irradiation (2 Gy) and examined p-ɣH2AX foci clearance in TOV112D and TOV1946 cells. As expected, an increased number of p-ɣH2AX foci were observed 1 h after radiation exposure, followed by rapid clearance. However, p-ɣH2AX persisted longer when Ran was knocked down (Fig. 2E). Next, we sought to determine whether Ran KD impaired the homologous recombination (HR) and non-homologous end-joining (NHEJ) DNA DSB repair systems. HR was evaluated by quantifying BRCA1 and Rad51 foci formation in dividing cells (Geminin-positive cells) and NHEJ was assessed by using a plasmid-based NHEJ repair reporter assay that relies on the ligation rate of digested I-SceI ends of a GFP-reporter vector [22]. Our results showed that Ran KD significantly decreased HR and NHEJ efficiencies in TOV112D, TOV1946, and OV886(2) cells (Fig. 2F–H). Furthermore, we found that Ran also influenced DNA single-strand break repair since the KD of this GTPase was accompanied by a substantial decrease in PARP activity (Fig. 2I). Overall, these data pinpointed the role of Ran in DDR and highlighted the implication of this GTPase in DNA repair processes.

To investigate the involvement of DNA damage accumulation in the sensitivity of aneuploid cells to Ran loss, we induced DNA damage in diploid ARPE and TOV81D cells by irradiation before assessing their sensitivity to Ran KD. As irradiation is well known to induce senescence in normal cells [23], we first evaluated the levels of senescence-associated β-galactosidase in diploid cells at different doses of irradiation (Supplementary Fig. S2C). We selected a dose of 0.5 Gy for subsequent experiments as it did not induce a senescent phenotype but significantly increased the number of p-ɣH2AX foci (Supplementary Fig. S2C, D). In agreement with our observation following aneuploidy induction (Fig. 1F and Supplementary Fig. S1G), irradiation-induced DNA damage in ARPE and TOV81D cell was accompanied by an increase in active Ran-GTP and RCC1 levels (Fig. 2J). Furthermore, as observed for TOV112D and TOV1946 cells, we found that Ran KD in irradiated ARPE and TOV81D cells delayed p-ɣH2AX foci clearance of these diploid cells (Supplementary Fig. S2D). Interestingly, induction of DNA damage sensitized ARPE and TOV81D cells to the loss of Ran (Fig. 2K, L). Overall, our data demonstrate that the sensitivity of aneuploid EOC cells to Ran KD is at least partly due to the involvement of Ran in the DDR process.

### Ran/NR1D1 axis is crucial for aneuploid EOC cell survival

**Fig. 3:**
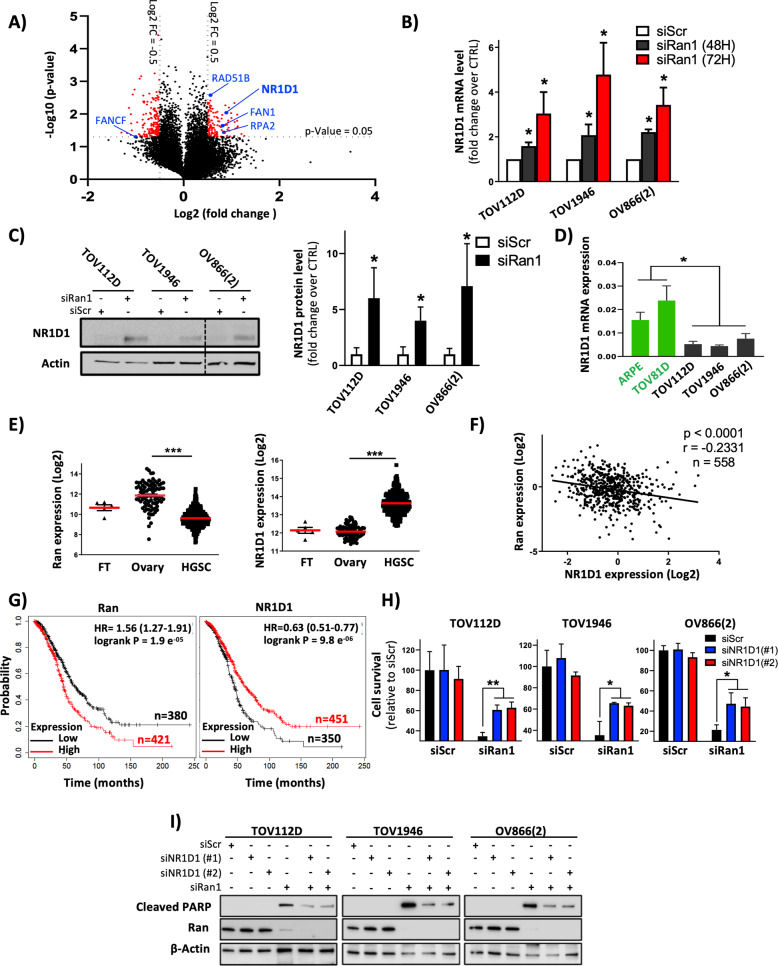
Ran/NR1D1 axis is crucial for aneuploid EOC cell survival. **A** Volcano plot representing gene variation following Ran KD. Significant genes (*P* value < 0.05) with a log2 fold change more than 0.5 are represented as red dots. Among these genes, those that have a known function in DNA repair are presented as blue dots. **B**, **C** TOV112D, TOV1946, and OV866(2) cells were transfected with siRan1 before analyzing the expression of NR1D1 by qRT-PCT (**B**) and western blot (**C**). **D** Expression of NR1D1 was evaluated by RT-PCR in ARPE, TOV81D, TOV112D, TOV1946, and OV866(2) cells. **E** The expression of Ran and NR1D1 in normal ovary, fallopian tubes, and HGSC was extracted from Xenabrowser website. **F** Inverse correlation between gene expression levels of Ran and NR1D1 using the TCGA dataset of HGSC tumors (*n* = 558). **G** Prognostic value of Ran (Affymetrix probe ID: 200750_s_at) and NR1D1 (Affymetrix probe ID: 31637_s_at) in ovarian cancer patients with optimal debulking was obtained using Kaplan–Meier plotter (www.kmplot.com). **H**, **I** TOV112D, TOV1946, and OV866(2) cells were transfected with siRan1 together with siRNAs targeting NR1D1 before assessing cell survival (**H**) and apoptosis (**I**). *P < 0.05, **P < 0.01, ***P < 0.001 (*n* = 3, Student’s *t*-test).

To better understand the molecular mechanisms linking Ran to DNA repair, gene expression microarray experiments were carried out using three aneuploid EOC cell lines [TOV112D, TOV1946, and OV866(2)] that were either transfected with siScr or siRan1. For our analysis, we focused on genes that were significantly up- or downregulated by a log2 fold change of more than 0.5 (Fig. 3A). We found that five of these genes were closely related to DNA repair (see blue dots on the volcano plot) and among them, NR1D1 was the most affected DNA repair gene induced by Ran KD (Fig. 3A). The nuclear receptor *NR1D1*, one of the circadian clock genes [16, 18, 19], has been recently shown to play a pivotal role in DNA repair [18, 24], particularly regarding the HR, NHEJ, and PARP activities that we found were also affected following Ran KD. Furthermore, NR1D1 has been shown to harbor tumor suppressor potential and as observed with Ran KD, its activation has been shown to display a differential sensitivity between normal and transformed cells [16, 18, 19]. Based on these data, we decided to focus on the link between Ran and NR1D1. To validate the data generated from the microarray study, we confirmed the upregulation of NR1D1 by real-time PCR (Fig. 3B and Supplementary Fig. S3A) and western blot (Fig. 3C and Supplementary Fig. S3B) in TOV112D, TOV1946, and OV866(2) cells transfected with siRan1 or siRan2. Next, we sought to determine whether the induction of NR1D1 following Ran KD was biologically relevant. To do this, we investigated the expression of the core circadian component, BMAL1 that is negatively regulated by NR1D1 [25]. Our data showed that Ran KD significantly inhibited the expression of BMAL1 confirming the relevance of NR1D1 induction (Supplementary Fig. S3C). Interestingly, our real-time PCR experiments also revealed that basal levels of NR1D1 expression in diploid cells were significantly higher than that of aneuploid EOC cells (Fig. 3D). In order to strengthen our in vitro observations regarding the link between Ran and NR1D1, we investigated the expression of these two genes in patient samples using publicly available data sets. When interrogating the Xena Functional Genomics Explorer (https://xenabrowser.net), we found that the expressions of Ran and NR1D1 were respectively up- and downregulated in HGSC specimens when compared to that of normal ovary or fallopian tubes (Fig. 3E). Furthermore, using the gene expression microarray data from 558 HGSC patients available through the TCGA Network (Cancer Genome Atlas Research, 2011), we established a significant Pearson’s “inverse” correlation between Ran and NR1D1 expression (*r* = −0.2331, *P* < 0.0001) (Fig. 3F). In our previous work using samples from ovarian cancer patients, we have shown that Ran expression was correlated with poor prognosis [8, 10, 26]. This prompted us to investigate the clinical relevance of NR1D1. Using the publicly available KM plotter tool (http://kmplot.com), we confirmed the prognostic value of Ran and demonstrated for the first time that the expression of NR1D1 was associated with prolonged survival of patients with ovarian cancer (Fig. 3G). Overall, these findings highlight the link between Ran and the tumor suppressor NR1D1 and support the relevance of this axis.

To test whether Ran signals through NR1D1 and if disruption of this axis is responsible for the sensitivity of aneuploid EOC cells upon loss of Ran, we decided to perform a double KD of Ran and NR1D1. For the NR1D1 KD, two siRNAs were used (siNR1D1 #1 or #2) (Supplementary Fig. S3D, E). Our results show that NR1D1 KD in TOV112D, TOV1946, and OV866(2) partly rescued the effect of Ran KD on cell survival (Fig. 3H) and apoptosis (Fig. 3I). Taken together, these results demonstrate for the first time that Ran regulates the expression of the tumor suppressor NR1D1 and that the Ran/NR1D1 axis is crucial for aneuploid EOC cell survival.

### Ran/NR1D1 axis is involved in DNA repair

**Fig. 4:**
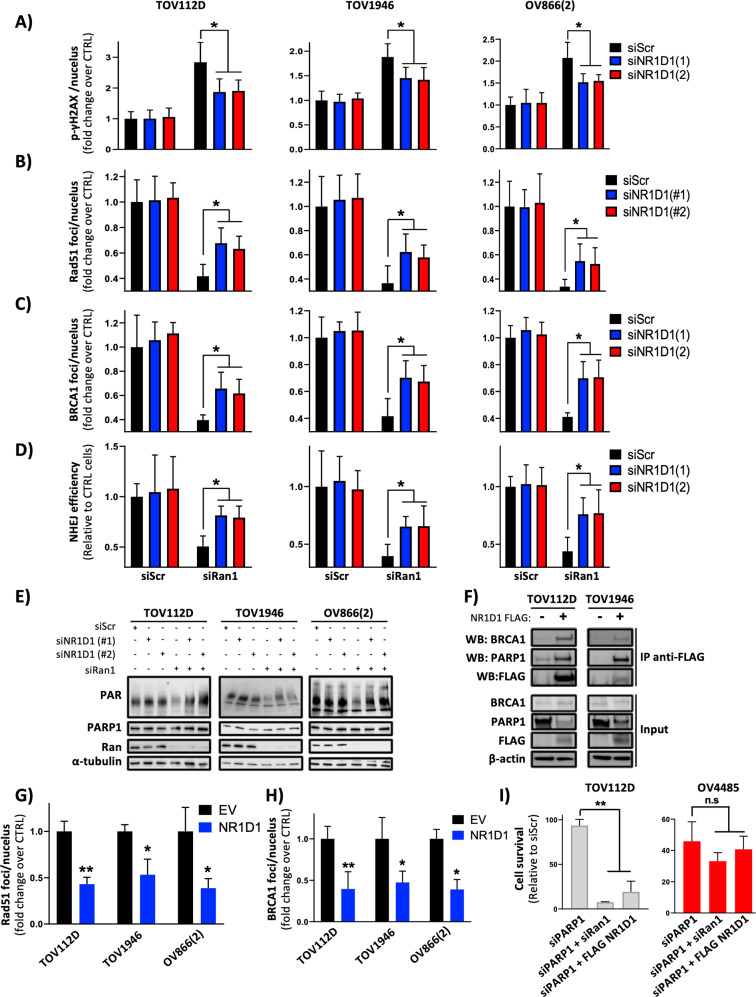
Ran/NR1D1 axis is involved in DNA repair. **A**–**E** TOV112D, TOV1946, and OV866(2) cells were transfected with siRan1 together with siRNAs targeting NR1D1 before analyzing p-γH2AX (**A**), Rad51 (**B**), and BRCA1 (**C**) foci formation; NHEJ efficiency (**D**) and PARP activity (**E**). **F** TOV112D and TOV1946 cells were transfected with a plasmid coding FLAG-NR1D1. Forty-eight hour after transfection, co-IP experiments were performed in the protein extracts using an anti-FLAG antibody. The resultant precipitates were subjected to western blot analysis using PARP1 and BRCA1 antibodies. **G**, **H** Cells were transfected with FLAG-NR1D1 plasmid or empty vector (EV) and treated with Olaparib (10 µM) then Rad51 and BRCA1 immunostaining was performed. Quantitative analysis of Rad51 (**G**) and BRCA1 (**H**) foci are shown. **I** TOV112D (left) and OV4485 (right) cells were transfected with siPARP1 together with siRan1 or FLAG-NR1D1 in order to assess cell survival using the IncuCyte live cell monitoring system. *P < 0.05, **P < 0.01, ***P < 0.001 (*n* ≥ 3, Student’s *t*-test).

To evaluate whether the induction of NR1D1 reproduced the effects of Ran KD regarding DNA repair, we overexpressed this nuclear receptor in TOV112D, TOV1946, and OV866(2) cells and we assessed the efficiency of the HR (Supplementary Fig. S4A, B), NHEJ (Supplementary Fig. S4C), and PARP (Supplementary Fig. S4D) pathways. We observed that these systems were significantly repressed following NR1D1 overexpression (Supplementary Fig. S4A–D). Furthermore, as we have shown following Ran KD (Fig. 2K), irradiated ARPE and TOV81D became significantly more sensitive to NR1D1 overexpression than non-irradiated counterparts (Supplementary Fig. S4E). Importantly, NR1D1 silencing mitigated the effect of Ran KD regarding DNA damage accumulation (Fig. 4A), the HR (Fig. 4B, C), and NHEJ (Fig. 4D) pathways as well as PARP activity (Fig. 4E). Collectively, these data demonstrate that the regulation of DNA repair by Ran is mediated partly by NR1D1. It has been reported that the modulation of DNA repair by NR1D1 comes from its ability to physically interact with PARP1 leading to the inhibition of PARP1 activity and the parylation of NR1D1. Parylated NR1D1 is then recruited to DNA damage sites where it impedes the recruitment of DDR components involved in the HR and NHEJ systems [18, 24]. Thus, it was suggested that the regulation of the HR and the NHEJ pathways by NR1D1 is PARP1 dependent. To verify whether PARP1 is a prerequisite for the modulation of DNA repair by NR1D1 in EOC, we performed co-immunoprecipitation assays in TOV112D and TOV1946 cells and we confirmed the interaction between NR1D1 and PARP1 (Fig. 4F). Moreover, we found that even in the presence of a PARP inhibitor (PARPi) (Olaparib), NR1D1 efficiently inhibited the induction of Rad51 (Fig. 4G) and BRCA1 (Fig. 4H) foci formation. This suggests that the HR regulation by NR1D1 occurs independently from PARP1 and argues for a new mechanism by which NR1D1 may regulate DNA repair. Interestingly, our co-immunoprecipitation experiments revealed that besides PARP1, NR1D1 interacts with BRCA1 (Fig. 4F). This finding together with the observation that NR1D1 overexpression inhibits BRCA1 foci formation (Fig. 4H and Supplementary Fig. S4B) indicates that the regulation of the HR pathway by NR1D1 occurs at least partly through a direct interaction with BRCA1. Moreover, our data suggest that NR1D1 overexpression (or Ran KD) represses simultaneously BRCA1 and PARP1 pathways. These results are of interest because it has been established that the inhibition of these two proteins alone has little effect on cell survival; however, when combined, they become lethal for cancer cells. To investigate the synthetic lethality of aneuploid EOC through modulation of the Ran/NR1D1 axis, we selected TOV112D (BRCA1^WT^) and OV4485 (BRCA1^−/−^) cell lines for further investigation. As expected, PARP1 KD has little effect on TOV112D cells but shows a significant toxicity in OV4485 cells (Supplementary Fig. S4F, G) confirming the synthetic lethality in the context of PARP1 and BRCA1 targeting. Furthermore, as shown in Fig. 1B and Supplementary Fig. S4H, both cell lines were sensitive to Ran KD and NR1D1 overexpression, which is in alignment with a simultaneous inhibition of PARP1 and BRCA1 pathways. Importantly, neither Ran KD nor NR1D1 overexpression accentuated the effect of PARP1 KD in the OV4485 cell line (Fig. 4I). However, when the same experiment was performed in TOV112D cells, Ran KD and NR1D1 overexpression potentiated the effect of PARP1 KD (Fig. 4I). Collectively, these data strongly suggest the importance of the Ran/NR1D1 axis in maintaining EOC cell survival through the modulation of PARP1 and BRCA1 pathways.

### Ran modulates the expression of NR1D1 through miR4472 maturation

**Fig. 5:**
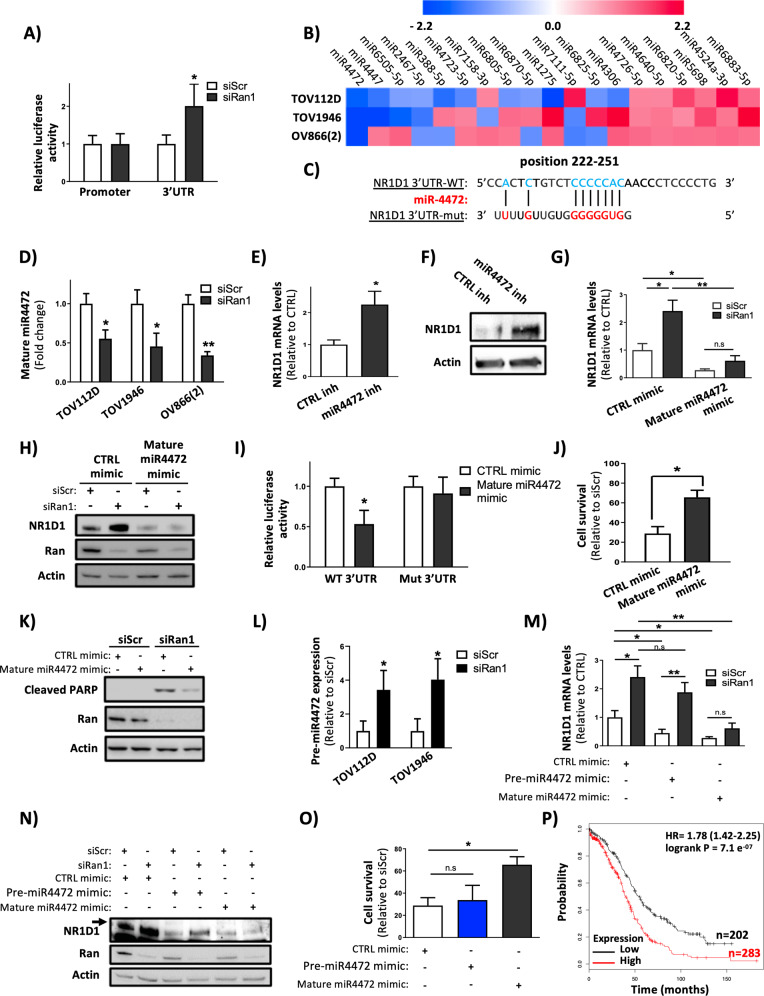
Ran regulates NR1D1 expression through miR4472 maturation. **A** Luciferase activity was measured in TOV112D cells transfected with siScr or siRan1 together with luciferase reporters expressing the promoter or the 3’UTR of NR1D1 mRNA. The luciferase activity was normalized with protein concentration. **B** miRNA qPCR-based array of the selected 19 miRNAs was performed in TOV112D and TOV1946 cells transfected with siRan1 to assess the expression of 19 miRNAs identified by miRDB databases as potential regulators of NR1D1. The heatmap shows the expression ratio between siRan- and siScr-transfected cells. **C** The predicted binding sequences for miR4472 within the WT and mutated human NR1D1 3’UTR. Seed sequences are highlighted. **D** Validation by RT-PCR in biological triplicate (from TOV112D, TOV1946, and OV866(2) cells) of results shown in (**B**) confirming the downregulation of miR4472 following Ran KD. **E**, **F** Cells were transfected with inhibitors of miR4472 to assess the expression of NR1D1 by RT-PCR (**E**) and western blot (**F**). **G**, **H** TOV112D cells were transfected with a mature miR4472 mimic together with siRan1 to assess the expression of NR1D1 by RT-PCR (**G**) and western blot (**H**). **I** Luciferase activity was measured in TOV112D cells transfected with miRcont or the mature miR4472 mimic together with luciferase reporters expressing the WT or mutated 3’UTR of NR1D1 mRNA. The luciferase activity was normalized to protein concentration. **J**, **K** TOV112D cells were treated as in (**G**) and (**H**) to assess cell survival using the IncuCyte live cell monitoring system (**J**) and apoptosis by western blot using cleaved PARP antibody (**K**). **L**–**N** TOV112D cells were transfected with pre-miR4472 or mature miR4472 mimics, together with either siScr or siRan1, to assess the expression of NR1D1 by RT-PCR (**L**) and western blot (**M**), as well as the cell survival relative to siScr using the IncuCyte live cell monitoring system (**N**). **O** TOV112D and TOV1946 cells were transfected with siScr or siRan1 and the expression of pre-miR4472 was evaluated by RT-PCR. **P** Prognostic value of miR4472 in ovarian cancer was obtained using Kaplan–Meier plotter (www.kmplot.com). *P < 0.05, **P < 0.01 (*n* ≥ 3, Student’s *t*-test).

To decipher the mechanism by which Ran KD induces the expression of NR1D1, we used a luciferase reporter plasmid containing either the promoter or the 3’UTR region of NR1D1. Ran KD in TOV112D cells had no effect in the expression of the former reporter; however, a significant increase of luciferase activity was observed when cells were transfected with the latter reporter (Fig. 5A). This suggests that NR1D1 increase following loss of Ran is mediated by miRNA dysregulation. In an attempt to identify miRNAs that may regulate NR1D1 expression, we used miRDB databases (http://mirdb.org/miRDB/) and established a list of 19 putative miRNAs recognizing NR1D1 mRNA. The expression of these miRNAs was evaluated following Ran KD in TOV112D, TOV1946, and OV866(2) cells using a miRNA qPCR-based array of the selected 19 miRNAs (Genecopoeia) (Fig. 5B). Among the dysregulated miRNAs, we focused on miR4472 (Fig. 5C) since it was downregulated following Ran KD in all three cell lines studied (Fig. 5B). We validated our miRNA array data involving miR4472 by RT-PCR (Fig. 5D). By using miRNA inhibitors targeting miR4472 (Supplementary Fig. S5A), we showed that NR1D1 was induced in TOV112D cells at the mRNA (Fig. 5E) and the protein (Fig. 5F) levels. In addition, the overexpression of a miR4472 mimic (mature form) (Supplementary Fig. S5B) decreased NR1D1 expression (Fig. 5G, H, siScr conditions). These data show that miR4472 regulates the expression of NR1D1. To determine whether this regulation occurs by a direct binding of the miRNA to the 3’UTR region of NR1D1 mRNA, we used a modified NR1D1 3’UTR reporter plasmid mutated in the predicted motif recognized by miR4472 (Fig. 5C). Our results show that overexpression of miR4472 inhibited the luciferase activity of the WT construction, but had no effect when the mutated reporter was used (Fig. 5I). Collectively, these data provide strong arguments for a direct role of miR4472 in NR1D1 suppression. Importantly, we found that overexpression of miR4472 partially reverses the hallmarks of Ran KD in TOV112D cells in regard to NR1D1 induction (Fig. 5G, H), cell survival (Fig. 5J), and apoptosis (Fig. 5K). Together, our data support the notion that the regulation of NR1D1 expression by Ran is, at least in part, mediated by miR4472. We next investigated whether Ran is involved in the expression or the maturation of miR4472. To this end, we evaluated the expression of the precursor of miR4472 (pre-miR4472). We found that the inhibition of the mature form of miR4472 following Ran KD (Fig. 5D) was accompanied by an accumulation of pre-miR4472 in TOV112D and TOV1946 cells (Fig. 5L) indicating that Ran is involved in the maturation of miR4472. To strengthen this finding, we compared the overexpression of a pre-miR4472 mimic with that of a mature miR4472 mimic. We found that these two miR4472 mimics were able to inhibit NR1D1 expression in the presence of Ran (Fig. 5M, N). However, only the mature miR4472 mimic was able to prevent NR1D1 induction and cell death caused by the loss of Ran (Fig. 5M–O). Collectively, our data demonstrate that Ran regulates miR4472 at the maturation level.

To our knowledge this is the first study attributing a biological activity to miR4472. Based on our finding showing a prognostic value of Ran and NR1D1 in the context of ovarian cancer (Fig. 3G), we investigated whether this miRNA was correlated with patient survival using the KM plotter tool. Interestingly, we found that ovarian cancer patients having high miR4472 levels showed significantly poorer overall survival (Fig. 5P). These data demonstrate for the first time the potential clinical relevance of miR4472.

### The relevance of Ran/NR1D1 axis in vivo

**Fig. 6:**
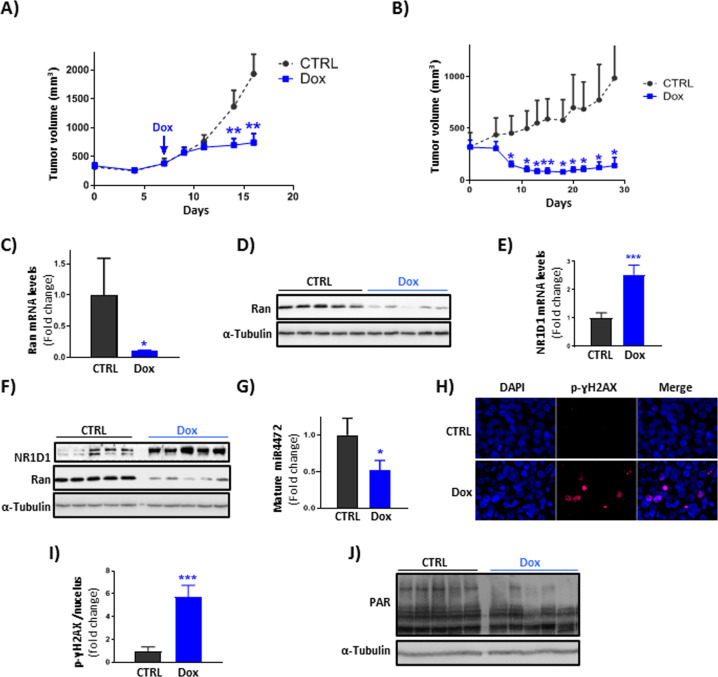
The relevance of Ran/NR1D1 axis in vivo. **A**, **B** TOV112D-Tet-shRan (**A**) and TOV1946-Tet-shRan (**B**) cells were subcutaneously injected in NRG mice and tumor growth was measured three times a week. Doxycycline (Dox)-supplemented food inducing shRNA expression started when tumors reached approximately a volume of 400 mm^3^. **C**–**G** Tumors from (**A**) were used for RNA and protein extraction. Ran (**C**, **D**) and NR1D1 (**E**, **F**) expression levels were assessed by RT-PCR (**C**, **E**) and western blot (**D**, **F**). **G** The expression of miR4472 was evaluated by RT-PCR. **H** p-γH2AX staining (red) was performed in tumor sections from (**A**). Representative images obtained from each group are shown. Nuclei were stained by DAPI. **I** Quantitative analysis of p-γH2AX foci obtained from (**H**) is shown. **J** PARP activity was evaluated in tumors by western blot using an anti-PAR antibody. *P < 0.05, **P < 0.01, ***P < 0.001 (Student’s *t*-test).

Based on our in vitro data showing the relevance of Ran GTPase as a promising therapeutic target of EOCs, we next assessed the outcome of Ran targeting in vivo. We have previously generated clones of the aneuploid TOV112D and TOV1946 cells stably expressing tetracycline (Tet)-inducible shRan (Supplementary Fig. S6A) [9]. We subcutaneously injected these clones in NRG mice and when tumors reached a volume of approximately 400 mm^3^, doxycycline (Dox)-supplemented food was given to the experimental groups. Loss of Ran expression in the TOV112D-Tet-shRan xenograft-based model showed a dramatic delay in tumor growth as exemplified by a significant decrease in tumor volume (Fig. 6A) and weight (Supplementary Fig. S6B). In the TOV1946-Tet-shRan xenograft-based model, the effect of Ran loss was particularly impressive as tumors started to regress 5 days after initiating Dox consumption (Fig. 6B) and treated mice survived significantly longer than controls (Supplementary Fig. S6C). However, by the end of the experiment tumors started to re-grow. As we have previously shown [9], this observation is most likely due to a selective pressure that results in the outgrowth of cells that are able to escape Ran depletion. Due to the availability of biological samples, only the TOV112D-Tet-shRan xenograft tumors were used to study, at the molecular level, the effects of loss of Ran in vivo. Our RT-PCR and western blot analyses showed a significant decrease of Ran expression at the mRNA (Fig. 6C) and protein (Fig. 6D) levels, demonstrating the effectiveness of our model to KD Ran in vivo. Significantly, we observed that Ran loss was accompanied by NR1D1 induction at the mRNA (Fig. 6E) and protein (Fig. 6F) levels together with a decrease of mature miR4472 (Fig. 6G). Furthermore, consistent with the involvement of Ran/NR1D1 axis in the DDR observed in vitro, our data show that tumors lacking Ran displayed a significant accumulation of DNA damage, exemplified by an increase in the number p-ɣH2AX foci (Fig. 6H, I), and a decrease in PARP activity (Fig. 6J). Taken together, our in vivo study reproduced the molecular outcomes that we observed in vitro following Ran KD, and argue for the importance of the Ran/NR1D1 axis in aneuploid EOC survival.

## Discussion

Many cancers are characterized by centrosome abnormalities and widespread aneuploidy [27, 28]. In this study, we have shown that targeting Ran is effective to eliminate aneuploid cancer cell lines, including those that are resistant to conventional chemotherapy treatments, with no or little effect in diploid cancerous or normal cell lines. These data extend the results of a previous study in which the authors observed by in vitro nuclear fusion (chromosomal gain) of a normal human fibroblast cell line, the establishment of steep Ran-GTP gradients comparable to that of the aneuploid Hela cells [29]. However, the authors did not investigate the sensitivity of these cells to Ran KD. Here we showed that genomic instability and aneuploidy can trigger an increase in Ran activity and sensitivity to the loss of Ran. Moreover, the use of a diploid cancer cell line in this study allowed us to provide evidence demonstrating that aneuploidy, rather than cell transformation, is behind the high dependency of cells on Ran activity. Therefore, our findings change the concept that Ran KD differentially affects normal and cancer cells [11, 12], and demonstrate that it is rather diploid versus aneuploid cells.

Our transcriptomic study has revealed a link between Ran and one of the components of circadian rhythm, NR1D1. This observation was further confirmed as we established, using TCGA data of patients with HGSC, a significant inverse correlation between the expression of Ran and NR1D1. Mechanistically, we showed that Ran is involved in the maturation of miR4472 that, in turn, regulates NR1D1 expression through the destabilization of its coding mRNA. It is known that Ran together with exportin 5 mediates the transport of pre-miRNAs from the nucleus to the cytoplasm where they are processed giving rise to functional mature miRNAs [30]. Thus, it is likely that owing to its role in the nuclear export process, Ran participates in miR4472 maturation. Since the re-expression of miR4472 rescued partly the effect of Ran KD regarding the expression of NR1D1, it is possible that this regulation also involves other actors. In fact, we have shown that other putative miRNAs recognizing the 3’UTR region of NR1D1 are under the control of Ran. Therefore, it would be interesting in future studies to investigate the role of these miRNAs in NR1D1 regulation.

A growing interest in studying circadian rhythm in the context of cancer has risen [17, 23] and pharmacological modulation of circadian components can be lethal for cancer cells [19]. NR1D1 has also been reported as a tumor suppressor gene [16, 17]. In this study we showed that overexpression of NR1D1 reproduced the effects of Ran depletion, while NR1D1 silencing partially rescued cells from the observed DNA damage accumulation following loss of Ran. One possible explanation for this partial rescue is that we focused only on the Ran/NR1D1 axis. However, as a key player in nucleo-cytoplasmic trafficking, Ran also contributes to DDR by recruiting 53BP1 (a key enzyme involved in DNA DSB repair) to DNA damage sites [31].

Although most of the literature on NR1D1 has been centered on its role as a transcriptional repressor in circadian rhythm, lipid metabolism, and inflammation [13–15], only one group has shown that NR1D1 regulates DNA repair at the post-translational level in breast cancer models [18, 24]. They found that the ligand-binding domain of NR1D1 interacted with DNA binding domain of PARP1 inhibiting PARP1 activity as well as the HR and NHEJ pathways. Based on the findings that a mutated form of NR1D1 lacking the ligand-binding domain (328 amino acids) was unable to repress the HR and NHEJ pathways, and that PARPi treatment abrogated the recruitment of NR1D1 to DNA damage sites, the authors suggested that DNA repair regulation by NR1D1 is PARP1 dependent [24]. Here we provide strong data showing that NR1D1 is a DDR repressor in ovarian cancer cells as well, but that this regulation occurs through a direct interaction of NR1D1 with both PARP1 and BRCA1. Surprisingly, we showed that even in the presence of a PARPi, NR1D1 overexpression was able to significantly inhibit HR, suggesting that the regulation of DNA repair by NR1D1 in ovarian cancer is not solely dependent on PARP1. One possible explanation for this discrepancy is that the ablation of the large ligand-binding domain of NR1D1 might have disturbed its interaction with both PARP1 and BRCA1. Moreover, in the context of PARP1 inhibition, the interaction between NR1D1 and BRCA1 most probably occurs before the recruitment of BRCA1 to DNA damage sites explaining the decreased NR1D1 localization near DNA lesions. Although further studies are warranted, our work sheds light on a new mode of action of NR1D1 and suggests that a detailed investigation of the interactome of this nuclear receptor might identify novel pathways/partners that are regulated by NR1D1 at the post transcriptional level.

Recently, the treatment of HGSC patients harboring a *BRCA1* or *BRCA2* mutation has expanded to include the FDA-approved PARPi therapy [32]. Based on our findings showing that Ran KD or NR1D1 overexpression abrogated simultaneously PARP activity and the HR pathway, we anticipate that targeting Ran/NR1D1 axis in the context of monotherapy would be sufficient to induce lethality in HGSC cells, including those that are HR proficient. In this study, we tested different cell lines with different sensitivities to PARPi [e.g., TOV1946 sensitive, OV4485 BRCA1^–/–^ intermediate, OV866(2) resistant] [33] and showed that, irrespective to their genetic background or sensitivity to PARPi, Ran KD was lethal for all tested aneuploid EOC cell lines.

In conclusion, our study puts forward Ran GTPase as a very promising therapeutic target for HGSC, and by inference all cancers associated with aneuploidy, that should be considered for future studies. We therefore suggest a synthetic lethal strategy targeting aneuploid cells based on their dependency to Ran.

## Methods

The human EOC cell lines [TOV81D, TOV112D, TOV1946, OV866(2), OV1946, TOV2295(R), OV4485] were derived in our laboratory from patients’ tumors (TOV) or ascites (OV) and characterized in detail [34–38]. All EOC cell lines were maintained in a low oxygen condition of 7% O_2_ and 5% CO_2_ and grown in OSE medium (Wisent, Montreal, QC). The human retinal epithelial cell line ARPE was purchased from American Type Culture Collection (ARPE-19, #CRL2302) and maintained in DMEM-F12 (Wisent). All media were supplemented with 10% FBS (Wisent), 0.5 μg/mL amphotericin B (Wisent), and 50 μg/mL gentamicin (Life Technologies Inc). Antibodies, chemicals, oligonucleotides, plasmids, and commercial assays used in this study are indicated in Supplementary Table S1.

### IncuCyte cell proliferation phase-contrast imaging assay

For Ran KD experiments using siRNA, cells were seeded in a 96-well plate (4000 cells/well) directly after transfection. The next day (day 0) and 72 h later (day 3), cell confluence was imaged by phase contrast using the IncuCyte live cell monitoring system (Essen BioScience, Ann Arbor, MI). Frames were captured at the indicated times using a 10× objective. All experiments were performed in triplicate wells for each condition and repeated at least three times.

### Ran activation assay

Assays were performed using the Ran activation assay kit (Cell Biolabs) according to the manufacturer’s instructions.

### Microarray analysis

Cells were transfected with siRan1 or siScr and cultured for 48 h. RNA extraction was performed as described previously [33] and gene expression microarray experiments were performed at the McGill University and Genome Quebec Innovation Centre (genomequebec.mcgill.ca) using human-Clariom S arrays (Affymetrix^®^). The resulting data were normalized and analyzed using Expression Console (Affymetrix^®^) and Transcriptome Analysis Console (Affymetrix^®^) software, respectively. Gene expression values were used to calculate ratios of siRan/siScr for each cell line.

### In vivo study

TOV112D-Tet-shRan or TOV1946-Tet-shRan cells with Tet-inducible expression of shRan were previously generated in our laboratory [9]. Six-week-old female NRG mice (NOD-*Rag1*^*null*^ *IL2rg*^*null*^, NOD rag gamma) obtained from the Jackson laboratory (Bar Harbor, ME) were injected with 1 × 10^6^ TOV112D-Tet-shRan or TOV1946-Tet-shRan cells suspended in a mix of 1:1 PBS and matrigel (BD Biosciences) at subcutaneous sites. Once the tumors reached a volume of 400 mm^3^, mice were randomly divided into control diet- and Dox-supplemented food (625 mg/kg) (Harlan)-group (5 mice/group). Measurements of tumor size were collected at least twice a week until tumors reached limit points (2000 mm^3^). All animal experiments were conducted following accepted standards of animal care in accordance with our institutional committee on animal care (CIPA), including the number of animals used in each group.

Methods for oligonucleotide transfection, RT-PCR, western blot, flow cytometry, immunofluorescence, immunoprecipitation, luciferase assay, clonogenic assay, metaphase spread, NHEJ assay, senescence detection, induction of aneuploidy/tetraploidy, and statistical analysis are described in Supplementary Information.

## Supplementary information

Supplementary figures

Supplementary information

Supplementary Table S1

## Data Availability

All relevant data are available from the authors. The microarray dataset was deposited to ArrayExpress with accession number E-MTAB-10558.
