# Supplementary material for: NR1D1 regulation by Ran GTPase via miR4472 identifies an essential vulnerability linked to aneuploidy in ovarian cancer
Source: Oncogene. 2021 Nov 6;41(3):309–20. doi: 10.1038/s41388-021-02082-z (PMC8755527; doi:10.1038/s41388-021-02082-z)
Supplement: Supplementary file 1 — Supplementary figures [file 41388_2021_2082_MOESM1_ESM.docx]

**Figure S1:Ran KD sensitivity is associated with the ploidy state of EOC cells. (A)**Ran activity (Ran-GTP) was evaluated in EOC cells and normal ARPE cells using a Ran-GTP pull-down assay. Diploid cells in green. **(B)** Evaluation by Western blot ofRCC1 expression in the cell lines used in Figure 1.**(C)** Western blots showing the efficiency of siRan1 and siRan2 in ARPE and EOC cells. **(D)** EOC cells and normal ARPE cells were transfected with siRan2 and subjected to proliferation assays using an IncuCyte live cell monitoring system. Green and black bars represent diploid and aneuploid cells, respectively. Data are expressed as the percentage of siScr-transfected cells at the end of experiment (96 h post-transfection) and are representative of at least three independent experiments. **(E, F)** Cells were transfected with siRan2 in order to evaluate apoptosis (96 h post-transfection) by Western blot using an anti-cleaved PARP antibody **(E)** and by flow cytometry using an Annexin V fluorescent antibody **(F)**.The percentage of Annexin V-positive cells (means from three independent experiments) is shown.**(G)** The expression of RCC1 was evaluated by Western blot after induction of aneuploidy in ARPE and TOV81D cells by Nocodazole treatment. **(H)** Tetraploidy was induced in ARPE and TOV81D cells by cytochalasin D treatment and assessed by counting binucleated cells (upper panel) after immunofluorescence staining (lower panel). Blue: DAPI, Green: Alpha tubulin, Red: Gamma tubulin. **(I)** Tetraploidy was induced in ARPE and TOV81D cells prior to Ran KD with siRan1 and siRan2. Subsequently, cells were subjected to proliferation assays. 4N: Tetraploid cells.*P < 0.05, **P < 0.01 (n ≥ 3, Student's t-test). In **(D)**, t-tests compared the indicated cancer cells with ARPE cells. In **(F)**, t-tests were among siRan1-transfected cells, comparing indicated cancer cells with ARPE cells.

**Figure S2: Ran is involved in the DNA repair process. (A)**Normal and EOC cancer cells were synchronized by serum starvation before quantifying p-γH2AX foci **(B)** Quantitative analysis of p-γH2AX foci in normal and EOC cancer cells after Ran KD with siRan2. **(C)** ARPE cells were treated with increasing doses of irradiation, cultured for 24 h and then subjected to β-galactosidase staining. **(D)** ARPE and TOV81D cells were transfected with siScr or siRan1 and then exposed to 0.5 Gy X-rays. Cells were fixed at the indicated recovery time points and immunostained for p-γH2AX. Quantitative analysis of p-γH2AX foci is shown. *P < 0.05, **P< 0.01 (n = 3, Student's t-test). In **(A)**, t-tests compared the indicated cancer cells with ARPE cells. In **(C)**, t-tests compared irradiated cells with control cells.

**Figure S3: Ran negatively regulates NR1D1 expression. (A, B)** Cells were transfected with siRan2 for 72 hours before analyzing the expression of NR1D1 in TOV112D, TOV1946 and OV866(2) by qRT-PCT **(A)** and in TOV112D by Western blot **(B)**.**(C)** The expression of BMAL1 was analyzed by qRT-PCR in TOV112D, TOV1946 and OV866(2) cell lines following Ran KD. **(D, E)**The efficiency of siNR1D1 (#1) and siNR1D1 (#2) was analyzed by qRT-PCR **(D)** and Western blot **(E).** *P < 0.05, (n = 3, Student's t-test).

**Figure S4: NR1D1 is involved in DNA repair.**TOV112D, TOV1946 and OV866(2) cells were transfected with FLAG-NR1D1 plasmid or empty vector (EV) before analyzing Rad51 **(A)** and BRCA1 **(B)** foci formation, the NHEJ efficiency **(C)** and PARP activity **(D).** The experimental conditions were the same as in Figure 2F-I. **(E)** ARPE and TOV81D cells were transfected with FLAG-NR1D1 or EV plasmids then exposed to 0.5 Gy X-rays in order to assess cell survival using theIncuCyte live cell monitoring system. **(F)** TOV112D and OV4485 cells were transfected with siRan1 and/or siPARP1 and the expression of Ran and PARP1 was evaluated by Western blot 96 hours after transfection. **(G, H)** TOV112D and OV4485 cells were transfected with siPARP1 **(G)** or NR1D1 FLAG plasmid **(H)** in order to assess cell survival using the IncuCyte live cell-monitoring system. *P < 0.05, **P <0.01, ***P <0.001 (n ≥ 3, Student's t-test).

**Figure S5: miR4472 regulates the expression of NR1D1. (A-B)** TOV112D cells were transfected with inhibitors **(A)** or mature mimic of miR4472 **(B)** and the expression of the target miRNA was evaluated by RT-PCR.Data are expressed as the fold change over control cells. *P < 0.05, **P < 0.01 (n >= 3, Student's t-test).

**Figure S6: Ran is a potential therapeutic target of EOC. (A)** TOV112D-Tet-shRan and TOV1946-Tet-shRan cells were treated with doxycycline (Dox) for 72 hours and resultant protein extracts were subjected to Western blot to analyze the expression of Ran. **(B)** Tumor weight excised from control and Dox groups of TOV112D-Tet-shRan xenografts are shown. **(C)** Kaplan–Meiersurvival curves of mice used in Figure 6B. **p <0.01 (Student's t-test).
