# Supplementary material for: NR1D1 regulation by Ran GTPase via miR4472 identifies an essential vulnerability linked to aneuploidy in ovarian cancer
Source: Oncogene. 2021 Nov 6;41(3):309–20. doi: 10.1038/s41388-021-02082-z (PMC8755527; doi:10.1038/s41388-021-02082-z)
Supplement: Supplementary file 2 — Supplementary information [file 41388_2021_2082_MOESM2_ESM.docx]

**Supplementary information**

**Methods**

**Oligonucleotide transfection**

Suspensions of 10^6^ cells in 100 µL of nucleofector solution V (Lonza Group Ltd) were transfected by electroporation with 1.2 nmoles of the indicated siRNA or inhibitors/mimics of miRNA. Scramble siRNA or miRNA were used as controls in all the experiments.

**RT-PCR and western blot analysis**

For mRNA quantification, experiments were performed as described previously [1]. For miRNA quantification, RNA extraction was performed using MirVana miRNA Isolation Kit (Thermofisher). Reverse transcription of miRNAs and Pre-miRNAs was performed using All-in-One MiRNA Q-PCR Detection Kit (Genecopoeia) and High-Capacity cDNA Reverse Transcription Kit (Thermofisher), respectively.

**Apoptosis analysis by flow cytometry**

Cells were transfected with siRNAs and seeded in 6-well plates. Ninety-six hours after transfection, cells (floating and adherent) were collected and incubated for 30 minutes at room temperature with Annexin V antibody and 5 minutes at room temperature with DRAQ 7. A maximum of 30,000 events were counted per condition using the Fortessa flow cytometer (BD Biosciences) and analyzed with the FlowJo software.

**NHEJ efficiency analysis**

NHEJ efficiency was assessed using the reporter plasmid pCDNA-GFP NHEJ, a gift from Dr. Jean-Yves Masson (U. Laval, QC, Canada) [2, 3]. Briefly, this plasmid expresses a GFP gene which is separated from its promoter by a puromycin cassette. The cassette is flanked on either side by two I-SceI-restriction sites. Removal of the cassette by the I-SceI enzyme produces a specific DNA DSB which is repaired by the NHEJ system. Efficient repair of these DNA DSBs allows the expression of the GFP gene. Our experimental procedure was as follows: TOV112D cells were electroporated with siRan 1 for 24 hours and subsequently transfected using lipofectamine (Invitrogen) with the reporter plasmid together with pCMV-I-SceI and mCherry expression vectors. Forty-eight hours later, cells were trypsinized, and the number of GFP-positive and mCherry-positive cells were determined by flow cytometry.

**Immunofluorescence**

Cells grown on cover slips were washed in ice-cold PBS, fixed in formalin and permeabilized with 0.25% Triton X-100 (Sigma–Aldrich Inc.). After blocking (1% BSA and 4% FBS in PBS), coverslips were incubated with the anti p-γ-H2AX (Ser139) or anti-Rad51 together with Geminin primary antibodies and subsequently incubated with Cy-5 (for p-γ-H2AX and Rad51) and Alexa Fluor 488 (for Geminin) secondary antibodies. For p-γ-H2AX foci clearance, cells were transfected with the indicated siRNA for 48 hours and then gamma-irradiated at 2 Gy and fixed at 1 and 24 hours after irradiation. For Rad51 foci quantification, cells were transfected with the indicated siRNA for 72 hours then gamma-irradiated at 10 Gy and fixed at 1 hour after irradiation. For all experiments, coverslips were mounted onto slides using Prolong® Gold anti-fade reagent with DAPI (Life Technologies Inc.). Images were obtained using a Zeiss microscope (Zeiss observer Z1, Carl Zeiss,Jena, Germany). Automated analysis software from Zeiss (AxioVision™, Carl Zeiss) was used for foci counting. In each condition, γ-H2AX and Rad51 foci were quantified in roughly 400 nuclei.

**Induction of aneuploidy with nocodazole**

Diploid ARPE and TOV81D cells were treated overnight with nocodazole (500 nM), then rigorously washed and further incubated with complete medium for 24 hours. The next day, cells were transfected with the indicated siRNA to assess cell proliferation and apoptosis. For these experiments, induction of aneuploidy was verified by metaphase spread experiments as described below.

**Metaphase spread**

Cells were treated with 100 nM of nocodazole for 3 hours and collected. Cell pellets were resuspended in 5 ml ice-cold 0.56% KCl and incubated at room temperature for 6 minutes. Cells were fixed in a methanol:acetic acid (3:1) solution and dropped on a microscope slide. Slides were left at room temperature to dry for 1 hour and then coverslips were mounted with Prolong® Gold anti-fade reagent with DAPI (Life Technologies Inc.). For each experiment, chromosomes were counted in approximately 50 nuclei under a Zeiss fluorescence microscope.

**Induction of tetraploidy with cytochalasin D**

Diploid ARPE and TOV81D cells were treated with nocodazole (500 nM) overnight. After two washes with complete medium, cells were treated with cytochalasin D (2.5 µg/mL) for 6 hours, then washed again twice and incubated with fresh media overnight. Cells were then transfected with the indicated siRNA, and cell proliferation was measured using the IncuCyte system. For these experiments, the induction of tetraploidy was verified by immunofluorescence. Treated cells were fixed, permeabilized and stained with alpha tubulin antibody conjugated with FITC and DAPI. The number of binucleated cells was determined using a Zeiss microscope (Zeiss observer Z1).

**Luciferase assay**

TOV112D cells were co-transfected with 100 ng of pLightSwitch reporter GoClone plasmids (Active motif) together with siRan 1. Forty-eight hours after transfection, luciferase activity was quantified using the LightSwitch Luciferase Assay Kit (Active motif). Data were normalized using negative non-transfected control cells and also the protein content in each condition.

**Immunoprecipitation assay**

These experiments were performed using protein samples obtained from cells transfected with a plasmid coding for FLAG-NR1D1. Anti-Flag antibody was diluted in lysis buffer and incubated with Dynabeads® Protein G (Life Technologies) for 10 minutes at room temperature. After PBS washing, 350 μg of proteins were immunoprecipitated using the Dynabeads-antibody complexes overnight at 4°C under gentle agitation. After PBS washing, protein complexes were eluted by incubating the beads 10 minutes at 95°C with a loading buffer (50 mM Tris pH6.8, 10% SDS, 10% glycerol, β-mercapto-ethanol and bromophenol blue). Subsequently, eluted proteins were subjected to Western blot analysis.

**Clonogenic survival assay to measure carboplatin sensitivity**

Clonogenic assays were performed as previously described [1]. IC_50_ values were determined using Graph Pad Prism 5 software (GraphPad Software Inc., San Diego, CA).

**Senescence-associated β-galactosidase detection**

Senescence-associated β-galactosidase analyses were performed as previously described [4].

**Immunofluorescence on xenograft tumor tissue**

The tumors collected from the mice were fixed in formalin and embedded in paraffin. Tumors were sectioned at a thickness of 4 μm, mounted onto glass slides, and stained with p-ɣH2AX using the Ventana automated immunostaining system. Briefly, antigen retrieval was carried out with Cell Conditioning 1 (Ventana Medical System Inc.) prior to incubation with the primary antibody for 60 minutes at 37°C. On the bench, we performed 20 minute incubations with blocking solution (Dako, Agilent) followed by Cy-5 conjugated secondary antibody for 45 minutes at room temperature. To quench tissue auto-fluorescence, slides were incubated for 15 minutes at room temperature with a 0.1% (w/v) solution of Sudan Black in 70% ethanol. Finally, slides were mounted using Prolong® Gold anti-fade reagent with DAPI (Life Technologies Inc.) and p-ɣH2AX foci visualization and counting were performed as described in the Immunofluorescence section.

**Statistical analysis**

Statistical analyses were performed using GraphPad software 5. Data from at least three independent experiments are presented as the means ± SD. Comparisons were carried out using Student’s t-test, which was appropriate for most experimental designs as the data was normally distributed and the variance between groups that were being statistically compared was similar. Data from TCGA database (ovarian serous cystadenocarcinoma [TCGA, provisional] / mRNA expression z-score [microarray]) were verified for their normal distribution using the Kolmogorov-Smirnov test. The correlation between Ran and NR1D1 was established using two-tailed Pearson's correlation test. Comparisons of survival curves were performed using log-rank test. A p-value of less than 0.05 was considered statistically significant.

**REFERENCES**

1 Fleury H, Carmona E, Morin VG, Meunier L, Masson JY, Tonin PN *et al*. Cumulative defects in DNA repair pathways drive the PARP inhibitor response in high-grade serous epithelial ovarian cancer cell lines. *Oncotarget* 2017; 8: 40152-40168.

2 Xie A, Kwok A, Scully R. Role of mammalian Mre11 in classical and alternative nonhomologous end joining. *Nat Struct Mol Biol* 2009; 16: 814-818.

3 Krietsch J, Caron MC, Gagne JP, Ethier C, Vignard J, Vincent M *et al*. PARP activation regulates the RNA-binding protein NONO in the DNA damage response to DNA double-strand breaks. *Nucleic Acids Res* 2012; 40: 10287-10301.

4 Dimri GP, Lee X, Basile G, Acosta M, Scott G, Roskelley C *et al*. A biomarker that identifies senescent human cells in culture and in aging skin in vivo. *Proc Natl Acad Sci U S A* 1995; 92: 9363-9367.
