# Supplementary material for: NR1D1 regulation by Ran GTPase via miR4472 identifies an essential vulnerability linked to aneuploidy in ovarian cancer
Source: Oncogene. 2021 Nov 6;41(3):309–20. doi: 10.1038/s41388-021-02082-z (PMC8755527; doi:10.1038/s41388-021-02082-z)
Supplement: Supplementary file 3 — Supplementary Table S1 [file 41388_2021_2082_MOESM3_ESM.docx]

**Table S1:** Key reagents and resources used in the study.

| REAGENT or RESOURCE | SOURCE | IDENTIFIER |
| --- | --- | --- |
| Antibodies | | |
| Ran | Santa Cruz | sc-271376 |
| Ran | Abcam | ab53775 |
| Cleaved PARP | Cell Signaling Technology | #9541 |
| PAR | Trevigen® | 4335-MC-100 |
| NR1D1 | Abcam | ab174309 |
| γ-H2AX (Ser139) | EMD Millipore | 05-636 |
| Rad51 | Abcam | ab213 |
| Geminin | Proteintech | 10802-1-AP |
| α-Tubulin | Santa Cruz | SC-32293 |
| FITC conjugated α-Tubulin antibody | Sigma-Aldrich Inc | F2168 |
| BV421 Annexin V | BD Biosciences | 563973 |
| β-Actin | Abcam | Ab6276 |
| Chemicals and dyes | | |
| NR1D1 agonist | Cayman Chemical | SR9011 |
| NR1D1 antagonist | EMD Millipore | SR8278 |
| DRAQ 7 | Abcam | ab109202 |
| Critical Commercial Assays | | |
| Ran activation assay kit | Cell Biolabs | STA-409 |
| LightSwitch Luciferase Assay Kit | Active Motif | 32031 |
| RNeasy Mini Kit | Qiagen | 74104 |
| All-in-One MiRNA Q-PCR Detection Kit | GeneCopoeia | QP015 |
| High-Capacity cDNA Reverse Transcription Kit | Thermofisher | 4368814 |
| mirVana™ miRNA Isolation Kit | Thermofisher | AM1560 |
| Oligonucleotides | | |
| **Primers for mRNAs** | **Sense (5’-3’)** | **Antisense (5’-3’)** |
| Ran | GGTGGTACTGGAAAAACGACC | CCCAAGGTGGCTACATACTTCT |
| NR1D1 | TGGACTCCAACAACAACACAG | GATGGTGGGAAGTAGGTGGG |
| β-Actin | CATGTACGTTGCTATCCAGGC | CTCCTTAATGTCACGCACGAT |
| **Primers for miRNAs** | **Source** | **Identifier** |
| miR4447 | GeneCopoeia | HmiRQP2075 |
| miR1275 | GeneCopoeia | HmiRQP0118 |
| miR6825-5p | GeneCopoeia | HmiRQP3746 |
| miR338-5p | GeneCopoeia | HmiRQP0425 |
| miR4524a-3p | GeneCopoeia | HmiRQP2170 |
| miR4472 | GeneCopoeia | HmiRQP2106 |
| miR6805-5p | GeneCopoeia | HmiRQP3706 |
| miR4306 | GeneCopoeia | HmiRQP1554 |
| miR5698 | GeneCopoeia | HmiRQP2728 |
| miR4723-5p | GeneCopoeia | HmiRQP2369 |
| miR6870-5p | GeneCopoeia | HmiRQP3836 |
| miR7111-5p | GeneCopoeia | HmiRQP4303 |
| miR2467-5p | GeneCopoeia | HmiRQP2483 |
| miR4640-5p | GeneCopoeia | HmiRQP2231 |
| miR4726-5p | GeneCopoeia | HmiRQP2376 |
| miR6505-5p | GeneCopoeia | HmiRQP2955 |
| miR7158-3p | GeneCopoeia | HmiRQP4337 |
| miR6820-5p | GeneCopoeia | HmiRQP3736 |
| miR6883-5p | GeneCopoeia | HmiRQP3862 |
| SNORD44 | GeneCopoeia | HmiRQP9011 |
| Pre-miR4472 | Thermofisher | 4426961 (Hs04274563_s1) |
| **siRNA, miRNA inhibitors and mimics** | **Source** | **sequence (5’-3’)** |
| siRan 1 | Dharmacon | CUAGGAAGCUCAUUGGAGA |
| siRan 2 | Dharmacon | GAAAUUCGGUGGACUGAGAUU |
| siPARP1 | Cell signaling | #6304 |
| shRan | Barres *et al*, 2010 | CACCAGAAGAATCTTCAGTACTATTCGAAAATAGTACTGAAGATTCTTC |
| siScr | Dharmacon | UCACAACCUCCUAGAAAGAGUAGA |
| miR4447 inhibitor | GeneCopoeia | AAACAACAGCCCCCACC |
| miR4472 inhibitor | GeneCopoeia | AAAACAACACCCCCCACC |
| Cont inhibitor | GeneCopoeia | CmiR-AN0001-SN |
| mature miR4472 mimic | GeneCopoeia | HmiR-SN2106-SN |
| pre-miR4472 mimic | GeneCopoeia | HmiR1146-MR04 |
| Cont mimic | GeneCopoeia | CmiR-SN0001-SN/ CmiR0001-MR04 |
| **plasmids** | **Source** | **IDENTIFIER** |
| NHEJ reporter plasmid | Dr. Jean-Yves Masson laboratory | N/A |
| NR1D1 Promoter plasmid | Switchgear Genomics | S714604 |
| NR1D1 3’UTR plasmid | Switchgear Genomics | S801799 |
| Mutated NR1D1 3’UTR plasmid | Bio Basic inc | N/A |
| pCMV-I-SceI expression vector | Dr. Jean-Yves Masson laboratory | N/A |
| mCherry expression vector | Dr. Jean-Yves Masson laboratory | N/A |
| FLAG-NR1D1 expression vector | Addgene | #22745 |
| Software and Algorithms | | |
| Graphpad Prism 5 | Graphpad | www.graphpad.com |
| FlowJo | FlowJo LLC | https://www.flowjo.com |
| miRDB | Department of Radiation Oncology,  Washington University School of Medicine | http://mirdb.org/miRDB |
| AxioVision | Zeiss | www.zeiss.com |
| Expression Console | Thermofisher | www.thermofisher.com |
| Transcriptome Analysis Console (TAC) | Thermofisher | www.thermofisher.com |
| MultiExperiment Viewer software _4_8_1 | TM4 | mev.tm4.org |
